# Genomic region detection via Spatial Convex Clustering

**DOI:** 10.1371/journal.pone.0203007

**Published:** 2018-09-11

**Authors:** John Nagorski, Genevera I. Allen

**Affiliations:** 1 Department of Statistics, Rice University, Houston, TX, United States of America; 2 Department of Electrical and Computer Engineering, Rice University, Houston, TX, United States of America; 3 Jan and Dan Duncan Neurological Research Institute and Department of Pediatrics-Neurology, Baylor College of Medicine, Houston, TX, United States of America; Chuo University, JAPAN

## Abstract

Several modern genomic technologies, such as DNA-Methylation arrays, measure spatially registered probes that number in the hundreds of thousands across multiple chromosomes. The measured probes are by themselves less interesting scientifically; instead scientists seek to discover biologically interpretable genomic regions comprised of contiguous groups of probes which may act as biomarkers of disease or serve as a dimension-reducing pre-processing step for downstream analyses. In this paper, we introduce an unsupervised feature learning technique which maps technological units (probes) to biological units (genomic regions) that are common across all subjects. We use ideas from fusion penalties and convex clustering to introduce a method for Spatial Convex Clustering, or SpaCC. Our method is specifically tailored to detecting multi-subject regions of methylation, but we also test our approach on the well-studied problem of detecting segments of copy number variation. We formulate our method as a convex optimization problem, develop a massively parallelizable algorithm to find its solution, and introduce automated approaches for handling missing values and determining tuning parameters. Through simulation studies based on real methylation and copy number variation data, we show that SpaCC exhibits significant performance gains relative to existing methods. Finally, we illustrate SpaCC’s advantages as a pre-processing technique that reduces large-scale genomics data into a smaller number of genomic regions through several cancer epigenetics case studies on subtype discovery, network estimation, and epigenetic-wide association.

## Introduction

Modern genomic technologies take fine-grained measurements on human subjects that allow for increasingly individualized treatment options for various diseases. Several of these technologies capture genomic information at spatially registered locations on the DNA sequence; examples include point mutations, next generation sequencing, copy number variation, and the focus of this paper, DNA Methylation arrays which measure epigenetic variation. Here, the units returned by the technology, CpG sites, are not of primary interest to scientists. More important are regions of CpG sites whose cumulative impact affects gene function [1]. To this end, we introduce an unsupervised feature learning technique which maps technological units to biological units by coalescing probes into contiguous genomic regions that are common across multiple subjects.

Epigenetic technologies measure genetic aspects that affect gene regulation beyond gene expression and transcriptomics. One such example is DNA Methylation, which measures the addition of a methyl group to CpG sites creating 5-methylcytosine. High methylation levels (hypermethylation) have been shown to block gene transcription in cancer. Similarly, low methylation levels (hypomethylation), typically at a global level, have also been observed by cancer researchers [1]. Recent advances in whole genome bisulfite sequencing technology now yield methylation intensity measurements at hundreds of thousands of CpG sites across the genome [2]. The ratio of methylated intensity to total intensity, the so-called beta-value, is returned as a measure of the DNA methylation level at each site. Such technologies interrogate genomic regions (such as gene promoter regions) by taking measurements of multiple CpG sites in close spatial proximity; beta-values in these regions are often strongly correlated, indicating that they behave functionally as a unit [3]. These observations imply that probes which are close in genomic distance and which display similar methylation levels are better treated as functional units, or genomic regions. Previous work on region detection in the context of methylation has focused on differentially methylated region (DMR) discovery. Approaches in this area have utilized both smoothing techniques [4, 5] and the concept of linkage disequilibrium [6]. The task of DMR discovery is, however, an inherently supervised one. In this work, we focus on the unsupervised discovery of genomic regions for methylation data. We develop a method for grouping probes into genomic regions that leads to more interpretable scientific measurements, improves the performance of downstream analyses, and can thus serve as a dimensionality reducing pre-processing step for methylation data.

Another example of grouping spatial genomics data is the well-studied problem of copy number segmentation. Copy number variation (CNV) is one measure of structural variability in the genome that quantifies large scale insertions and deletions of genetic information across the DNA sequence. By utilizing array-CGH technology, structural differences relative to a reference sample are quantified as the log ratio of intensities at various loci across the genome. Such differences have been linked to various cancers such as breast and lung [7]. Similar to DNA methylation, copy number measurements at a particular loci are typically of lesser interest than regions of gain or loss, which signal large-scale amplification or deletions [8]. As such, the problem of Copy Number Segmentation has received much attention and numerous methods exist for this type of analysis in both the single subject [9–11] and multi-subject [12–14] setting. Most popular among existing approaches are numerous segmentation algorithms, collected in the popular R-packages CNTools and DNACopy [15, 16]. Due to both its well-studied nature and similarity to region detection in the case of methylation data, we consider the task of copy number segmentation as a benchmark. We show that our method both performs competitvely to the state of the art as well as eliminates the need for subjective choices concerning the number and extent of detected segments.

**Fig 1:**
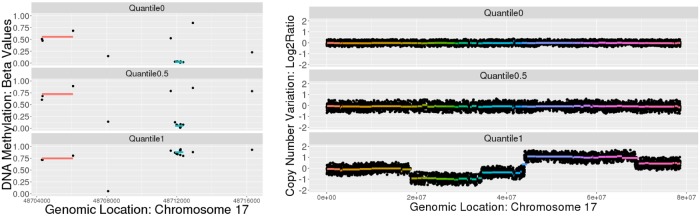
SpaCC detected regions for TCGA level 3 breast cancer DNA-methylation data *n* = 791, *p* = 23515 (left) and TCGA level 2 Ovarian Cancer copy number variation (array-CGH) data with *n* = 456, *p* = 7658 (right), both for chromosome 17. For each data type we plot raw probe values overlayed with SpaCC clusters for three subjects. Subjects are ordered according to maximum deviation from the average cluster centroid over all subjects. For methylation data we note the ordering detects a hypomethylated region (blue), while for copy number data the ordering sorts patients according to genetic variability. In both cases, we note the discovery of common genomic regions across all patients.

For both methylation and CNV data, scientists seek entire regions of CNV amplifications or deletions or regions of CpG sites that behave as functional units. For these regions to serve as meaningful features for downstream multivariate statistical analyses, they must be consistent across all subjects. To address this, we introduce an unsupervised learning method which maps technological units to more meaningful biological units by clustering probes into genomic regions. An example of the genomic regions resulting from our method, Spatial Convex Clustering (SpaCC), can be seen in Fig 1. To detect such regions, we utilize the popular concept of fusion penalties [17] by extending methods for convex clustering [18–23] Convex clustering has shown several advantages relative to traditional clustering methods, most notably its improved solution stability [19]. Additionally, by couching the clustering problem as a regularized convex optimization problem, convex clustering has allowed for numerous principled extentions into related domains such as biclustering [24] and clustering in high dimensions [25]. In a similar fashion, we introduce SpaCC which yields an extention of the convex clustering problem appropriate for spatial genomics data. We specifically focus on developing a fully automated and data-driven method that can be used to pre-process spatial genomics data into genomic regions for downstream analyses (Section Spatial Convex Clustering). While we illustrate our method on CNV data to benchmark our method against widely used segmentation approaches (Section Simulation Studies: Copy Number Segmentation), the main focus of this paper is on methylation data. For this, we show how our method can lead to improved results for biomarker discovery in region-based epigenetic-wide association studies (Section Region-Based Epigenetic-Wide Association Studies), and our resulting genomic regions can yield improved features in downstream multivariate analysis such as clustering for subtype discovery (Section Breast Cancer Methylation Subtype Discovery) and epigenetic network estimation (Section Inferring Epigenetic Networks).

## Spatial Convex Clustering

Our objective is to discover groups of probes which (i) have similar measurements, (ii) are spatially contiguous, and (iii) are nearby on the chromosome. Taken together, (i)-(iii) deliver groups of probes similar enough to be considered as single functional units, or genomic regions. Importantly, we require that (i)-(iii) be satisfied simultaneously for all subjects, so that the genomic regions discovered are intrinsically meaningful, and not artifacts of a particular sample. One approach to multi-subject genomic region detection would be to consider clustering the genomic probes based on the subject observations, as opposed to the more commonly used clustering of observations. To this end, we merge ideas that use fusion penalties for copy number segmentation [11, 14, 26] and the more recently introduced convex clustering methods [18, 19] to develop an automated pipeline for feature learning with spatial genomics data. In particular, we address data-specific weighting schemes, automated methods for detecting the number and extent of clusters, as well as a principled approach for handling missing values.

### Optimization problem and algorithm

Let $X\in {\mathbb{R}}^{n\times p}$ be our data matrix with *n* subjects and *p* variables (probes). Our SpaCC problem is defined as

$$\begin{array}{c} \begin{array}{cccc} & \underset{U\in R^{n\times p}}{\text{minimize}} & & \frac{1}{2}{∥X-U∥}_{F}^{2}+\gamma \sum_{i=1}^{p-1}w_{i}{∥U_{•i}-U_{•i+1}∥}_{2} \end{array} \end{array}$$ (1)

In the objective above we seek an estimate, $U\in {\mathbb{R}}^{n\times p}$, which both faithful to the original data (smooth loss term given by the Frobenius norm) and which encourages fusions among similar, adjacent probes (non-smooth *l*_2_ penalty term). The amount of fusion of among probes is determined by the size of regularization parameter *γ* ≥ 0 and the weights *w*_*i*_ ≥ 0. At one extreme, with *γ* = 0, SpaCC simply returns the original data matrix, ***X***, and no fusions or genomic regions are detected. As regularization increases (larger *γ*), columns difference ‖***U***_•*i*_ − ***U***_•*i*+1_‖ are forced to **0**, implying that ***U***_•*i*_ = ***U***_•*i*+1_. In the case of SpaCC, when such equality occurs we say that probe *i* and *i* + 1 have been clustered together and belong to the same genomic region. While the amount of regularization plays a key role in determining the clustering solution, weight choices are also important. As discussed subsequently, the latter are taken as spatial weights proportional to the inverse distance between adjacent probes that are specific to each genomic technology; these in turn, lead to more interpretable results and computationally efficient algorithms.

The SpaCC problem can be seen as a particular instance of the convex clustering problem, with several key differences allowing for its use with spatially-registered genomics data. First, convex clustering typically clusters observations (here rows of ***X***), whereas SpaCC clusters measurements so as to detect genomic regions. In this sense SpaCC can be viewed a performing convex clustering on ***X***^*T*^. Additionally, convex clustering allows fusions to occur among any pair of observations via the more general penalty term *P*(***U***) = *γ*∑_*i*<*j*_ *w*_*ij*_‖***U***_•*i*_ − ***U***_•*j*_‖ We note that such generality is not appropriate in the case of spatial data. As an example, employing the traditional convex clustering penalty in the case of SpaCC could result in non-contiguous genomic regions which make little sense in practice. By enforcing fusions only among adjacent probes, SpaCC’s augmented penalty properply accounts for the known spatial structure. In this sense, our method builds on the success of several existing fusion-based approaches that have been proposed specifically for CNV data [14, 26]. Finally, note that we apply our SpaCC problem separately to data for each chromosome.

To fit our SpaCC model, we adopt an approach introduced by [19] for convex clustering problems. We reformulate (1) by introducing an auxiliary variable ***V***, where ***V***_•,*i*_ = ***U***_•*i*_ − ***U***_•*i*+1_, and rewrite the penalty in terms of ***V***. We then use the Alternating Minimization Algorithm (AMA) [27] optimization algorithm to fit our SpaCC problem. Formulating the augmented Lagrangian, we obtain updates for both primal variables (***U***_•*i*_, ***V***_•*i*_) and dual variables (**Λ**_•*i*_), as shown in Algorithm 1.

**Algorithm 1**: SpaCC AMA Algorithm

**Data**: ***X***, ***w***, *γ*

**Result**: ***U***, ***V***

**while** *err* > *tol* **do**

$U_{•,i}^{k+1}=\left\{ \begin{array}{cc} X_{•1}+\Lambda_{•1}^{k} & i=1\\ X_{•i}+\Lambda_{•i}^{k}-\Lambda_{•i-1} & i\in \left\{2,\ldots ,p-1\right\}\\ X_{•p}-\Lambda_{•p-1}^{k} & i=p \end{array}\right.$

$V_{•,i}^{k+1}={\left[1-\frac{\gamma w_{i}/\nu }{\|U_{•i}^{k+1}-U_{•i+1}^{k+1}-\frac{1}{\nu }\Lambda_{•i}^{k+1}{\|}_{2}}\right]}_{+}\left(U_{•i}^{k+1}-U_{•i+1}^{k+1}-\frac{1}{\nu }\Lambda_{•i}^{k+1}\right)$

$\Lambda_{•,i}^{k+1}=\Lambda_{•i}^{k}+\nu (V_{•i}^{k+1}-U_{•i}^{k+1}+U_{•i+1}^{k+1})$

**end**

### Spatial weights

An important input to our SpaCC problem, and convex clustering generally, is the weight vector, ***w***. Two common weight choices have been made throughout the convex clustering literature [18, 19, 25]: uniform weights, with *w*_*ij*_ = 1, and distance-based weights. Uniform weights offer a potential methodological benefit in that they do not assume prior knowledge regarding potential fusions. In particular, no fusions are given apriori preference over others, as all are given equal weight. Uniform weights, however, suffer from two major disadvantages. The first disadvantage is computational. Distance-based weights allow for the incorporation of sparsity among the weights, greatly reducing computation time [19]; such a possibility is necessarily excluded in the case of uniform weights. Second, while a case for uniform weights may be made in the absence of domain-knowledge, when domain knowledge is present its incorporation via proper weight choices can deliver improved clustering solutions. In the case of spatial genomics data, such prior knowledge is especially evident. In particular, SpaCC utilizes both the spatial orientation of the probes, along with prior biological knowledge concerning measurement similarity decay to inform its weight choices. While uniform and distance-based weights are typical throughout the convex clustering literature, alternatives have been considered. In [25], for example, the authors consider an adaptive lasso [28] inspired weighting scheme, used to induce sparsity among the feature set. In the case of SpaCC, however, sparsity within the set of spatially contiguous probes is structurally undesirable, and we therefore utilize the distance-based approach.

For SpaCC, the choice of weights is inversely proportional to the genomic distance between adjacent probes, *w*_*i*_ = exp{−*σd*_*i*_}, where *d*_*i*_ = dist(probe_*i*_, probe_*i*+1_) is the distance in basepairs between probes. Our choices regarding the spatial decay parameter, *σ*, have been made to reflect the empirical differences between copy number variation and methylation data. In the the case of copy number variation data, probe measurements are often similar across sizable portions of the chromosome [29]. Hence, spatially expansive genomic regions (associated with the slower decay rate of *σ* = 0.00001) reflect the underlying biology. In the case of methylation measurements, probe similarity dissipates much more rapidly, with methylated regions forming in small localized CpG islands near promoter regions of genes [3]. For this data type, enforcing unnaturally slow decay rates can overshadow probe dissimilarity, resulting in genomic regions which share little in common. Hence for methylation data, we allow for faster decay (*σ* = 0.0002). More generally, the choice of *σ* will be based on both the empirical properties of the data as well as knowledge of the underlying phenomenon; in present case, examples of these weight choices are shown in S1 Appendix We demonstrate the efficacy of our choices for *σ* through both simulations, Section Simulation Studies, and real data examples, Section Applications of SpaCC to Cancer Genomics Data. Overall, tailoring spatial weights to specific technologies yields more interpretable genomic regions, with larger clusters in CNV data and smaller localized methylated regions, Fig 1.

Additionally, our choice of weights can dramatically decrease the computational burden of our SpaCC algorithm. Specifically, we apply hard-thresholding to the weights to set tiny weights to zero. Exact sparsity in the weight values prevents distant adjacent probes from coalescing into a common genomic region. Further if *T* weights are set to zero, the penalty term of (1) is perfectly separable into *T* + 1 terms, yielding *T* + 1 subproblems. These subproblems may in turn be solved in parallel. For example using our methylation weight choice, the Chromosome 17 TCGA Level 3 Breast Cancer Methylation SpaCC problem, Fig 1, separates into 910 subproblems that can be solved completely in parallel. This yields dramatic computational gains when compared to the original problem which consists of *p* = 23515 probes.

### Missing values and parameter selection

We seek to develop a fully automated method for detecting multi-subject genomic regions in CNV and methylation data; often automated methods are more reliable and reproducible, as practitioners have no knobs to tune that can yield different results across studies and labs. To this end, we introduce two automated, optimization-based approaches to handling practical problems with spatial genomics data: missing data and regularization parameter selection. First, missing values tend to be a major problem for large-scale high-throughput genomics data. For example, the TCGA Breast Cancer Level 3 Methylation Chromosome 17 data shown in Fig 1, contains 14270 total missing values. Similarly for TCGA Ovarian Cancer Level 2 Copy Number Variation, Chromosome 17 contains 2349 missing values. Typically, missing genomics data is handled via a two-step process where one first imputes the missing values using many popular off-the-shelf imputation routines [30] and then continues with analyses of the fully imputed data. This approach, however, is less reproducible as results of downstream analyses can change depending on the imputation procedure employed. Instead, we propose to fit our SpaCC model in the presence of missing data, effectively eliminating the need to preform a separate imputation step.

As before, let $X\in {\mathbb{R}}^{n\times p}$ be our data matrix. Let $ℳ={ℳ}_{n}\times {ℳ}_{p}\subset \left\{1,\ldots ,n\right\}\times \left\{1,\ldots ,p\right\}$ denote the indices of missing elements. We adopt an approach similar to that in [24] to fit our SpaCC procedure in the presence of missing values. Specifically, we fit our SpaCC loss function only over the the non-missing elements of ***X***, given by the indices ${ℳ}^{C}$:

$$\begin{array}{c} \underset{U\in R^{n\times p}}{\text{minimize}}\frac{1}{2}\sum_{j\in M_{p}^{C}}\sum_{i\in M_{n}^{C}}{\left(X_{ij}-U_{ij}\right)}^{2}+\gamma \sum_{i=1}^{p-1}w_{i}{∥U_{•i}-U_{•i+1}∥}_{2}. \end{array}$$ (2)

First, notice that this optimization problem is still convex, and hence our approach will yield the global solution. We propose to optimize (2) using the majorization minimization (MM) algorithm [31]. Defining the surrogate function to be

$$\begin{array}{c} \begin{array}{c} g\left(U|U^{k}\right)=\frac{1}{2}[\sum_{j\in M_{p}^{C}}\sum_{i\in M_{n}^{C}}{\left(X_{ij}-U_{ij}\right)}^{2}+\sum_{j\in M_{p}}\sum_{i\in M_{n}}{\left(U_{ij}-U_{ij}^{k}\right)}^{2}]\\ +\gamma \sum_{i=1}^{p-1}w_{i}{∥U_{•i}-U_{•i+1}∥}_{2}, \end{array} \end{array}$$ (3)

notice that *g*() majorizes the objective *f*(***U***); namely (i) *g*(***U*** ∣ ***U***^*k*^) ≥ *f*(***U***) for all ***U*** and (ii) *g*(***U***^*k*^ ∣ ***U***^*k*^) = *f*(***U***^*k*^). Iteratively minimizing the surrogate objective creates a non-increasing sequence of objective values *f*(***U***^*k*^). Defining $T=X_{{ℳ}^{C}}+U_{ℳ}^{k}$, we augment (3) leading to following algorithm to fit our SpaCC problem in the presence of missing data:

**Algorithm 2**: SpaCC Algorithm for Missing Data

**Data**: ***X***, ***w***, *γ*, M

**Result**: ***U***, ***V***

**while** *err* > *tol* **do**

 Set $T=X_{M^{C}}+U_{M}^{k}$

 ***U***^*k*+1^ ← Alg. 1 at ***T***

**end**

Notice that in contrast to traditional imputation routines, Algorithm 2 does not explicitly replace missing elements in ***X*** prior to analysis. Rather, missing indices are filled in iteratively via the SpaCC cluster means for the genomic regions, ***U***.

Now that we have an automated way of fitting SpaCC with missing values, we leverage this to propose a *k*-fold cross-validation scheme to select the single tuning parameter, *γ*. Note that *γ* controls both the number of genomic regions and the extent of these regions. To select *γ*, we employ the approach of [32] where we remove random elements of ***X*** in each fold and take the optimal *γ* as the parameter whose SpaCC imputed solution fit in the presence of missing values most closely aligns with the removed data. Specifically, for k=1,…,K, where K the number of folds, we define the indices to be left out at the *k*th fold to be $C^{k}=C_{n}^{k}\times C_{p}^{k}\subset M_{n}^{C}\times M_{p}^{C}$, where ${⋃}_{k=1}^{K}C^{k}=M^{C}$, $C^{k}\cap C^{k\prime }=\emptyset$ and $|C^{k}|\approx \frac{1}{K}|M^{C}|$, so that ${\left\{C^{k}\right\}}_{k=1}^{K}$ is an approximately equal sized partition of the non-missing elements of ***X***. For each fold *k*, we introduce additional missing elements via $C^{k}$ and solve the missing data problem, Algorithm 2. Specifically, let $I^{k}=I_{n}\times I_{p}=(C_{n}^{k}\cup M_{n}^{k})\times (C_{p}^{k}\cup M_{p}^{k})$. Given *γ*, we solve SpaCC with missing values given by $I^{k}$:

$$\begin{array}{c} \underset{U\in R^{n\times p}}{\text{minimize}}\frac{1}{2}\sum_{j\in {\left(I_{p}^{k}\right)}^{C}}\sum_{i\in {\left(I_{n}^{k}\right)}^{C}}{\left(X_{ij}-U_{ij}\right)}^{2}+\gamma \sum_{i=1}^{p-1}w_{i}{∥U_{•i}-U_{•i+1}∥}_{2} \end{array}$$ (4)

We can solve this problem via Algorithm 2, replacing M with $I^{k}$; we denote the solution as $(U_{\gamma }^{*}{)}^{k}$. Then, we evaluate the performance of *γ* on the *k*th fold by comparing the solution $(U_{\gamma }^{*}{)}^{k}$ to the left out (non-missing) elements of ***X*** via mean squared error: $MSE(\gamma ,k)=\sum_{i\in C_{n}^{k}}\sum_{j\in C_{p}^{k}}{\left[(U_{\gamma }^{*}{)}_{ij}^{k}-X_{ij}\right]}^{2}$ Our complete cross validation algorithm is given as follows:

**Algorithm 3**: SpaCC Algorithm for Cross Validation

**Data**: ***X***, ***w***, *γ*, ${\left\{I^{k}\right\}}_{k=1}^{K}$

**Result**: ***U***, ***V***

**for** k=1,…K **do**

 **for** *γ* = *γ*_1_,…, *γ*_*T*_ **do**

  Algorithm 2 at λ with missing indices given by $I^{k}$

  Compute MSE over $C^{k}$

 **end**

**end**

**Fig 2:**
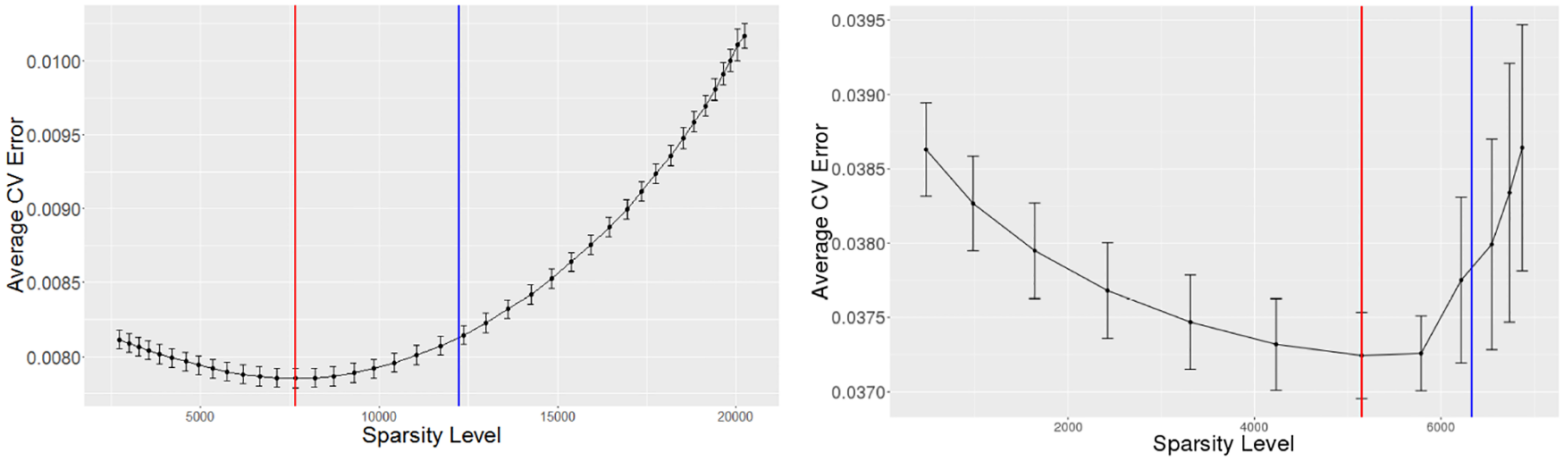
Cross validation plots for both methylation (left) and copy number (right). Sparsity-level is plotted along the x-axis and Average Error on the y-axis. The red line shows the sparsity-level of minimum Average Error, and the blue line shows sparsity level obtained after thresholding.

Given the tuning parameter, γ*, selected by minimizing the cross-validation error, we noticed that SpaCC identifies genomic regions which are spatially smaller than desired. Stated another way, cross-validation tends to underestimate the sparsity level in the differences, ***V***. Such behavior is well known for the lasso and other sparse problems and is hence why many advocate using the one-standard-error cross-validation rule [33]. An alternative approach is to post-process the results after cross-validation by thresholding [34]. We adopt such a scheme and propose to threshold the elements of ***V***_γ*_ at a level proportional to the estimated noise, ${\hat{\sigma }}^{2}$; specifically we threshold at the level, $|{\left(V_{\gamma^{*}}\right)}_{ij}|<\sqrt{\frac{\text{log}(p)}{n}}\hat{\sigma }$, similar to that proposed by [34]. Alternative thresholding procedures, for example block-wise thresholding, may also be employed. We note, however, that the element-wise method proposed here, is relatively more conservative in the sense of returning thresholded estimates similar to ***V***_γ*_. In Fig 2, we give examples of cross-validation error curves over a sequence of *γ* values for the TCGA Ovarian Copy Number and Methylation chromosome 17 example from Fig 1; both the minimum cross-validation error as well as our proposed thresholding level are shown. Overall, this approach delivers an automated, principled optimization-based method for handling missing data and selecting the number and extent of genomic regions. While the effectiveness of this method will be further studied in subsequent sections, we note at the outset that the automatic selection of the number of genomic regions offers a significant advantage over typical segmentation methods [12, 35]

## Simulation studies

We study the performance of SpaCC empirically and compare our approach to existing methods through simulations based on real array-CGH and DNA-methylation data. Before presenting our simulation results, it will be helpful to introduce some notation. Our probes are indexed by *j* ∈ {1, 2, …, *p*}. Associated with each probe is a genomic location $l_{j}\in \mathbb{R}$, *j* = 1, …, *p*, and we denote the distance between genomic locations by *d*_*ij*_. The probes are partitioned into clusters, *c*_*g*_, indexed by *g* = 1, …, *G*. For each probe *j* we denote the cluster to which it belongs via *r*(*j*) = {*g* ∣ *j* ∈ *c*_*g*_}. Our method and competitors will estimate a clustering ${\left\{{\hat{c}}_{g}\right\}}_{g=1}^{\hat{G}}$, with ${\hat{c}}_{g}\subset \left\{1,\ldots ,p\right\}$ and ${\hat{c}}_{g}\cap {\hat{c}}_{g^{\prime }}=\emptyset$. We will then evaluate the performance of all methods by comparing the estimated clustering ${\left\{{\hat{c}}_{g}\right\}}_{g=1}^{\hat{G}}$ to the true clustering ${\left\{c_{g}\right\}}_{g=1}^{G}$ using common clustering metrics such as the Rand, Adjusted Rand, and Jaccard Indexes [36]. Note that these clustering metrics are general and do not specifically account for the fact that we require clusters to be spatially contiguous. Hence, we also employ a lesser-known entropy-based clustering metric, the Variation of Information (VI) metric [37], that is better suited to measuring the information loss between two sets of clusters that may be nested, as we would often expect with spatially contiguous clusters; see S2 Appendix for details.

### Simulation studies: Copy number segmentation

We first evaluate the performance of SpaCC for the well-studied problem of copy number segmentation for array-CGH data. While not our primary focus, the problem shares many attributes in common with the problem of genomic region detection for methylation data. These common features along with many popular software packages [15, 16], employing segmentation methods such as Circular Binary Segmentation (CBS), make the problem an ideal benchmark to test the performance of our method.

Our simulations are based on TCGA Ovarian Cancer Level II array-CGH data for chromosome 17 [38], where we adopt the probe locations and use data for observed subjects to form the mean simulated CNV signal as well as the locations of copy number segments with amplifications or deletions. Specifically, we simulate series for *j* = 1…*p* probes and *i* = 1…*n* subjects from the following model: *X*_*ij*_ = *μ*_*j*_ + *s*_*i*,*r*(*j*)_*m*_*i*,*r*(*j*)_ + *ϵ*_*ij*_. Here, *μ*_*j*_ is the base mean of the series which is taken from a subject in the TCGA data that was detected as having no amplifications or deletions according to DNACopy [15]; *s*_*i*,*r*(*j*)_ is an indicator of an amplification or deletion for subject *i* in region *r*(*j*) where the regions *r*(*j*) are taken from the observed segments as detected by DNACopy for subjects from the TCGA data; *m*_*i*,*r*(*j*)_ gives the mean shift for the amplification or deletion in region *r*(*j*); and *ϵ*_*ij*_ ∼ *N*(0, *σ*) is iid additive noise. As not all subjects will have copy number changes for each region, we simulate *s*_*i*,*r*(*j*)_ ∼ *Bernoulli*(*q*). Also, as each subject could have an amplification or deletion of differing magnitude, we simulate *m*_*i*,*r*(*j*)_ ∼ ±*U*(*a*, *b*).

We study four simulation scenarios of varying difficulty. First, we study the effect of the magnitude of the copy number changes relative to the noise level by taking *a* = 0.2, *b* = 0.4, *σ* = .1 for large changes, and *a* = 0.05, *b* = 0.3, *σ* = .1 for small magnitude shifts that are more difficult to detect. Next, we use two different subsets of DNAcopy regions detected for a real TCGA patient, with easier or more difficult to detect shapes to seed the segment boundaries; that is, easier to detect segments tend to be spatially longer and harder to detect segments are shorter and more fragmented. Note that the easy and hard set of segments are shown in S5 Appendix. Also related to shape, we vary the probability of a shift, *q* = 0.7, 0.5, with a larger probability corresponding greater consensus across subjects, and hence easier shift detection.

The results in Table 1 indicate that SpaCC outperforms according to all metrics in all regimes in this simulation. As illustrated subsequently for real data, DNACopy segments each subject separately. As such, DNACopy performs well on per-subject basis, but performs poorly at the detecting segments common across all patients. To address this shortcoming, the CNTools package and its implementation of the Circular Binary Segmentation (CBS) algorithm offers an alternative whereby common segments are returned for all subjects. Yet, CNTools’ still fails to reach a reasonable consensus regarding common subject segments. Given the discrepancy between subject’s amplification/deletion regions, CNTools tends to deliver shorter segments, wherein agreement can be reached across several subjects. These short segments often partition the true, larger segments, but are both difficult to interpret and necessarily score poorly on the various metrics. In contrast, SpaCC’s regularized solution yields an improved consensus between the individual series and their common segments, detecting larger segments more closely aligned with the underlying truth.

**Table 1:** CNV segmentation performance of SpaCC, DNACopy, and CNTools over simulation regimes with easy or hard segment shapes (S) and easy or hard magnitude shifts (M).

	Rand			Jaccard			Variation of Information		
	SpaCC	DNACopy	CNTools	SpaCC	DNACopy	CNTools	SpaCC	DNACopy	CNTools
Easy(S)-Easy(M)	.98 (.03)	.87 (.02)	.94 (.02)	.94 (.09)	.76 (.03)	.77 (.12)	.06 (.11)	.38 (.05)	.43 (.18)
Easy(S)-Hard(M)	.99 (.02)	.88 (.02)	.93 (.02)	.97 (.06)	.76 (.03)	.71 (.11)	.02 (.07)	.39 (.05)	.63 (.19)
Hard(S)-Easy(M)	.99 (.00)	.89 (.01)	.85 (.02)	.99 (.01)	.73 (.03)	.38 (.09)	.00 (.02)	.39 (.04)	1 (.18)
Hard(S)-Hard(M)	.99 (.00)	.89 (.01)	.85 (.01)	.99 (.02)	.72 (.03)	.37 (.07)	.00 (.03)	.43 (.04)	1.15 (.17)

### Simulation studies: Methylation region detection

Next we evaluate SpaCC’s performance for the task of methylation region discovery. We again model our study based on real data, utilizing TCGA Breast Cancer Level 3 Methylation data for chromosome 17 [39]. Initial clusters, {*c*_*g*_}, are detected using SpaCC, which then act as the ground truth for what follows. We simulate methylation beta values via the cdf transform *X*_*ij*_ = Φ(*z*_*ij*_), for subjects *i* = 1, …, *n*, probes *j* = 1, …, *p*, and where Φ denotes the cdf of the standard normal distribution. Such a transformation ensures our simulated values to lie in (0, 1). Spatial correlation is introduced via the distribution of the *z*_*ij*_’s. Our simulated methylation regions are denoted by the degree of spatial correlation among probes within the region. Specifically, we take *z*_*ij*_ to be *z*_*i*_ ∼ *N*_*p*_(**0**, **Σ**). with spatial covariance given by $\Sigma_{ij}=\left\{ \begin{array}{cc} \text{exp}\left\{-d_{ij}/\sigma_{w}\right\} & r(i)=r(j)\\ \text{exp}\left\{-d_{ij}/\sigma_{b}\right\} & o.w. \end{array}\right.$ where *d*_*ij*_ is the distance between probes. Here *σ*_*w*_ controls the decay rate of the within cluster spatial correlation, and *σ*_*b*_ similarly between clusters. The difficulty of the simulation is controlled by difference between the the within and between spatial correlation decay rate. By varying the ratio of decay rates, *σ*_*b*_/*σ*_*w*_, we consider three scenarios of high, medium, and low within region spatial correlation, relative to between; these scenarios correspond to (*σ*_*w*_, *σ*_*b*_) = (100*KB*, .01*KB*), (100*KB*, .1*KB*), (100*KB*, 1*KB*), respectively. We compare SpaCC’s performance to the linkage disequilibrium method introduced in [6].

In Table 2 we again note that SpaCC outperforms existing region-detection methods across all regimes and metrics. A portion of SpaCC’s success may be attributed to the continuous nature of its spatial fusions, wherein clusters are joined in a smooth fashion via continuous spatial weight decay. The LD method, by contrast, implements a greedy strategy for accumulating clusters based on a discrete window size, here 500 bp. This fixed window size gives the LD method less flexibility to detect long range clusters when they are present; SpaCC, by not choosing apriori distance cutoffs, does not have this difficulty.

**Table 2:** SpaCC and linkage disequilibrium performance for region detection on simulated methylation data. We see SpaCC outperforms linkage disequilibrium across all metrics and for all levels of difficulty, given by the degree of spatial correlation.

	Variation of Information		Jaccard		Rand		AdjRand	
	SpaCC	LD	SpaCC	LD	SpaCC	LD	SpaCC	LD
High	.03 (.00)	.45 (.00)	.91 (.00)	.46 (.00)	.99 (.00)	.99 (.00)	.95 (.00)	.63 (.00)
Medium	.17 (.00)	.55 (.00)	.81 (.00)	.43 (.00)	.99 (.00)	.99 (.00)	.90 (.00)	.60 (.00)
Low	.52 (.02)	.76 (.00)	.60 (.04)	.37 (.00)	.99 (.00)	.99 (.00)	.75 (.03)	.54 (.00)

## Applications of SpaCC to Cancer Genomics Data

We now illustrate how SpaCC can be used as a pre-processing tool to improve the analysis of real array-CGH and DNA-methylation data. Using case studies from TCGA ovarian, breast, and lung cancers we show how SpaCC can yield improved copy number segmentation results in Section Ovarian Cancer Copy Number Segmentation, but mainly focus on the more novel application of detecting methylation regions. For this, we show how SpaCC can yield improvements in subtype discovery, Section Breast Cancer Methylation Subtype Discovery, inferring epigenetic networks, Section Inferring Epigenetic Networks, and biomarker discovery in region-based epigenetic-wide association studies (rEWAS), Section Region-Based Epigenetic-Wide Association Studies. As EWAS and especially rEWAS are relatively new types of epigenetic analyses, we include a more careful study of this application with additional simulation results in Section rEWAS Simulation Study and an application to TCGA lung cancer data in Section Lung Cancer rEWAS Study.

### Ovarian Cancer copy number segmentation

**Fig 3:**
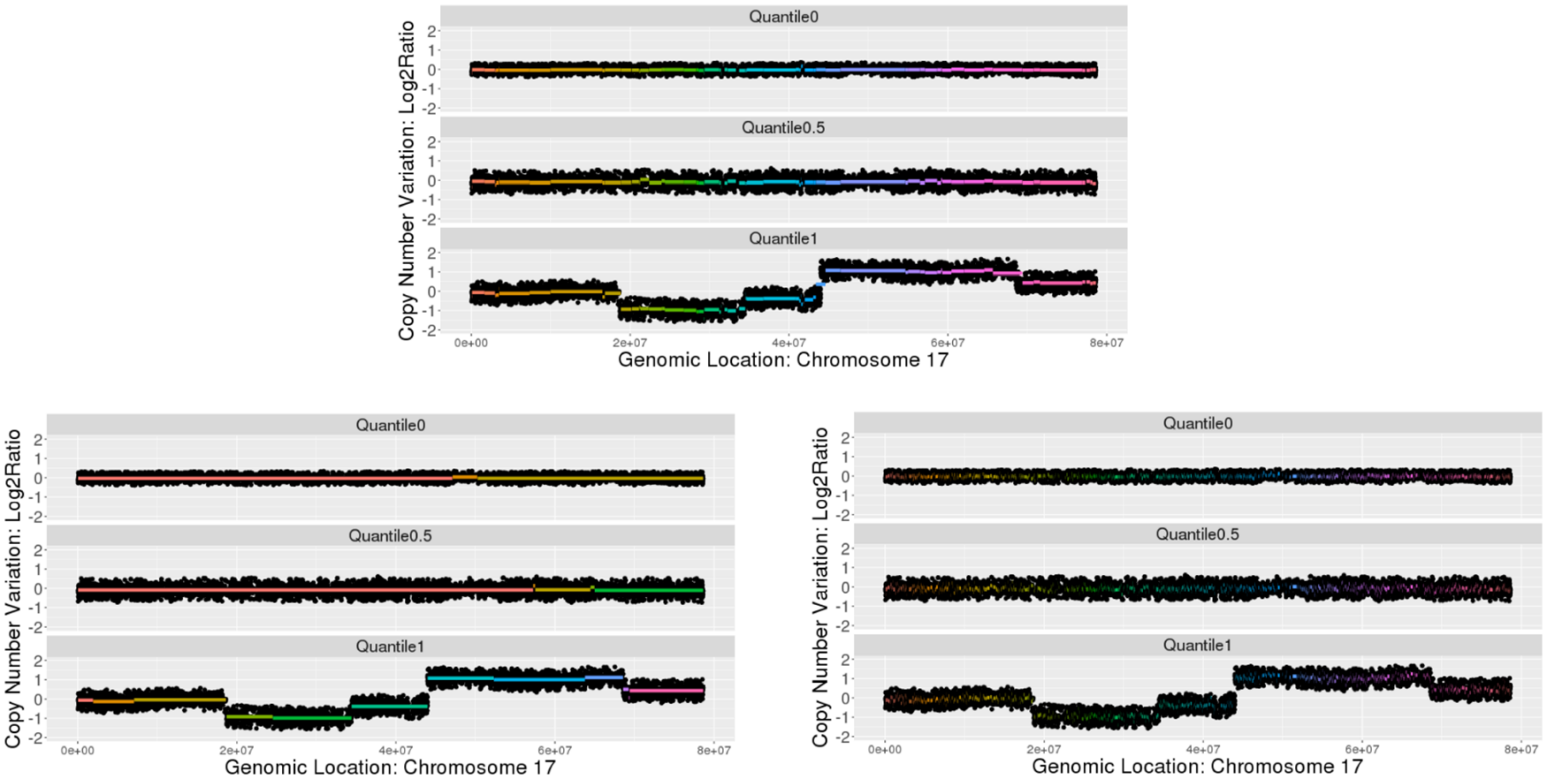
Copy number segmentation results for TCGA Ovarian Cancer chromosome 17. We compare SpaCC (top) to DNAcopy (bottom left) and CNTools (bottom right). Copy number series over the chromosome are represented by black points and segments are represented by horizontal colored lines. The subjects with the minimum, median, and maximum copy number changes over the cohort are visualized.

We investigate SpaCC’s segmentation performance on Ovarian Cancer TCGA Level 2 Copy Number data [38]. We report results for Chromosome 17, which is where the important BRCA1 gene, which has been widely associated with ovarian cancer [40], is located. The number of probes totals *p* = 7103 and we consider *n* = 456 subjects; SpaCC’s runtime in this case is 8 minutes and 30 seconds. In Fig 3, we visually compare segments discovered by SpaCC to those of the two most popular competitors, DNACopy [15] and CNTools [16]. We plot three subjects’ copy number series for the whole chromosome and overlay the estimated segments, colored consecutively, detected by each method; the level of each segment corresponds to the subject’s average copy number per segment. The three subjects plotted represent the subject with the minimum, median, and maximum copy number changes across the cohort.

These results visually illustrate the many advantages of SpaCC over existing tools for copy number segmentation. In particular, DNACopy, while performing well on a per-subject basis delivers inconsistent segments across multiple subjects. Indeed, the number of segments returned by DNACopy varies from 1 to 57, depending on subject. Thus, the segments produced by DNACopy cannot be used as a pre-processing step before further multivariate analyses, as the features are unaligned across the subjects. Conversely, CNTools, while delivering segments consistent across the subjects, finds difficulty assessing the trade-off between subject-specific patterns and patterns common to all subjects. The result is an awkward consensus with many (3754), short segments. This “shattered” appearance makes interpretation difficult due to both the number of segments and their size. In contrast, SpaCC finds longer more interpretable segments (61), finding a more appropriate balance between subject-specific and common patterns across the cohort. As such, SpaCC is ideally suited as a pre-processing method to segment and reduce the dimension of copy number data before further multivariate analyses.

### Detecting methylation regions with SpaCC

**Fig 4:**
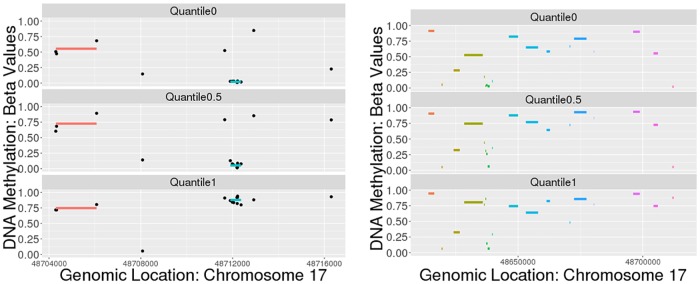
Breast cancer methylation region detection results. A portion of Chromosome 17 with SpaCC-detected regions overlayed for the subjects with the minimum, median, and maximum variation in methylation levels (left). We zoom in on the 48.70Mb–48.72Mb region of chromosome 17 (right) containing the promoter region of the ABCC3 gene.

We now study and illustrate how SpaCC can be used for biomarker discovery and as a pre-processing tool that yields a reduced and more meaningful set of features for downstream analysis of methylation data. First, we apply SpaCC to discover methylation regions from the TCGA Level 3 Breast Cancer data [39] for chromosome 17 with *n* = 791 subjects and *p* = 23515 probes. SpaCC’s runtime in the case of the methylation data is 18 minutes and 40 seconds. We visually examine methylation regions returned by SpaCC in Fig 4. Notice that the methylation regions detected are much shorter than their Copy Number counterparts. Segments in the latter tend to be amplified or deleted in large chromosomal regions, whereas regions of consistent methylation levels to occur in small localized areas corresponding to promoter regions of genes [3]. On the right in Fig 4, we can easily see how SpaCC features aggregate across probes whose levels are meaningful and consistent across all subjects; this is illustrated by the region denoted in blue. In contrast, SpaCC has not fused the two probes flanking this region as their levels differ significantly from the blue region for the subjects with the minimum and median variation in methylation levels shown. These local regions allow us to capture fine-grained characteristics that are more biologically meaningful than examining single probes. For example, the highlighted blue region is hypomethylated in most subjects, but hypermethylated for the bottom subject shown, which has the maximum variation in methylation levels. This region corresponds to the promoter region of the ABCC3 gene which is associated with HER2 and luminal breast tumors [41]. In total, SpaCC detected 9,080 methylation regions for chromosome 17, which offers a reduction from the original 23,515 probes.

#### Breast cancer methylation subtype discovery

**Fig 5:**
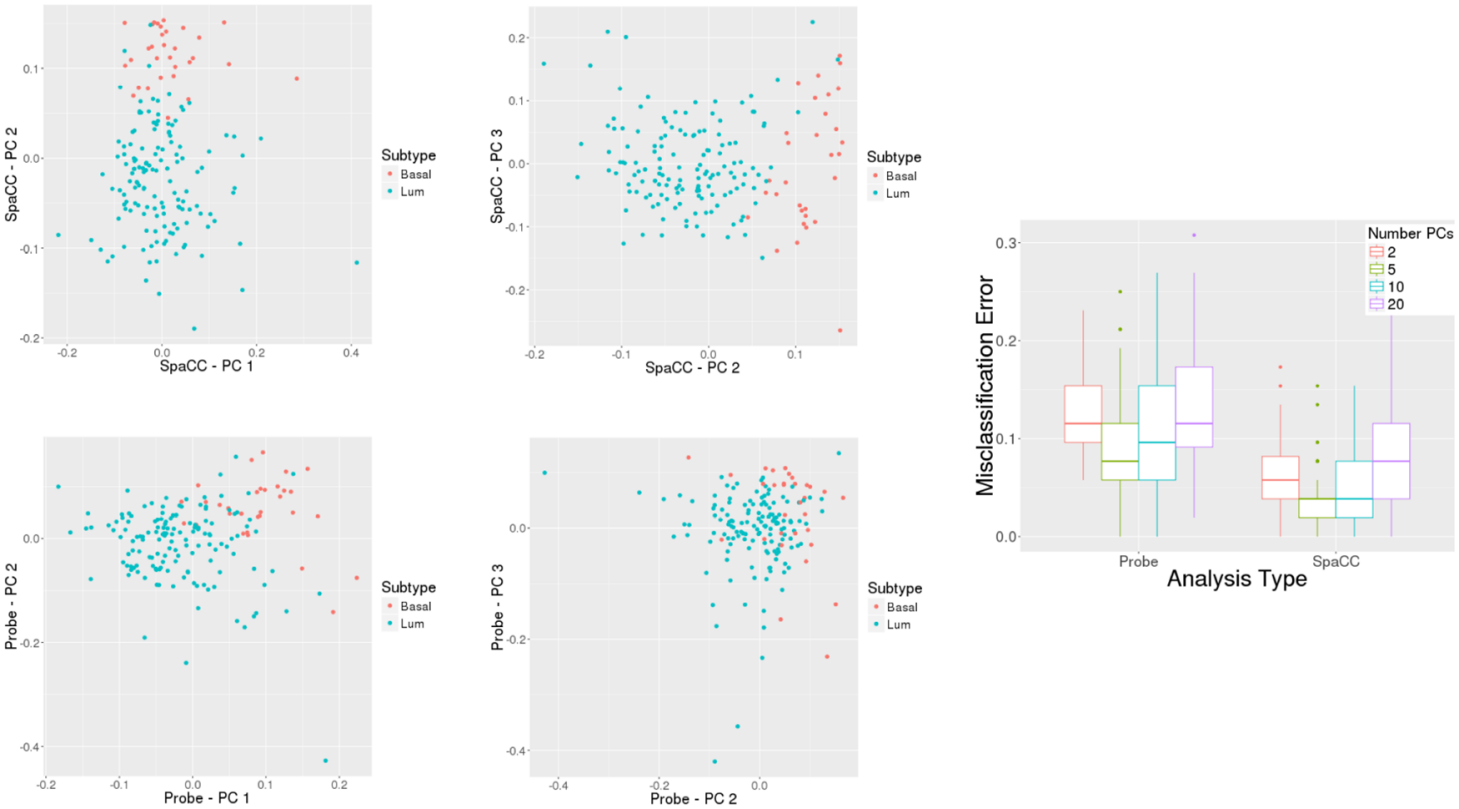
(Left) Principal Component scatterplots for SpaCC features (top) and raw probes (bottom), illustrating increased separation between Basal and Luminal subtypes for SpaCC features. (Right) Naive Bayes misclassification error on repeated test/train splits for both Probe and SpaCC features. SpaCC achieves lower error rates for all number of PC’s.

Several have recently suggested that methylation levels can be used to define cancer subtypes, and in breast cancer, methylation levels have been used to characterize the well-known expression-based subtypes [42]. Here, we illustrate how reducing methylation data to the SpaCC-derived reduced set of features offers improvements in downstream multivariate analyses such as subtype detection. We continue to work with the TCGA breast cancer data for chromosome 17 and consider classifying between the Basal and Luminal (A and B) subtypes. To fairly assess the performance of SpaCC’s region-based features relative to using the raw probe values, we consider a simple classification scheme where we first reduce the data using principal components and then fit a Naive Bayes classifier. In Fig 5, we repeatedly split the data into training and test sets and report the misclassification errors on the test sets for a range of principal components (PCs). We also show PC scatterplots for SpaCC-based and probe-based analysis, illustrating the superior discriminatory power of SpaCC-based features. Also notice that SpaCC-based features outperform in terms of misclassification error for all the number of PCs considered. By reducing the noisy probe-level data into more biologically meaningful units, SpaCC is able to yield improved results for subtype detection.

#### Inferring epigenetic networks

Various genomic regions have been consistently shown to be hyper- or hypomethylated together in sets of cancer patients [43]. One way of understanding relationships between methylated regions in cancer is by inferring epigenetic networks. Here, we show how SpaCC’s region-based features can lead to more meaningful and interpretable epigenetic networks. We continue to study the TCGA breast cancer chromosome 17 data and use Gaussian Graphical Models to infer networks using both raw probes and SpaCC-estimated regions. For the probe-based network, we consider the traditional approach, taking the raw probe measurements as nodes and representing conditional dependency estimates via connecting edges. For the region network, the mean vectors of SpaCC estimated genomic regions are used in place of raw probe vectors. As before, conditional dependencies, now between genomic regions, are represented via edges. In both cases, conditional dependency relationships are estimated via the graphical lasso [44, 45] with a common (dimension dependent) regularization parameter $\lambda =8\sqrt{\frac{\text{log}(p)}{n}}$ and stability selection [46] with a common threshold parameter of *τ* = .95.

**Fig 6:**
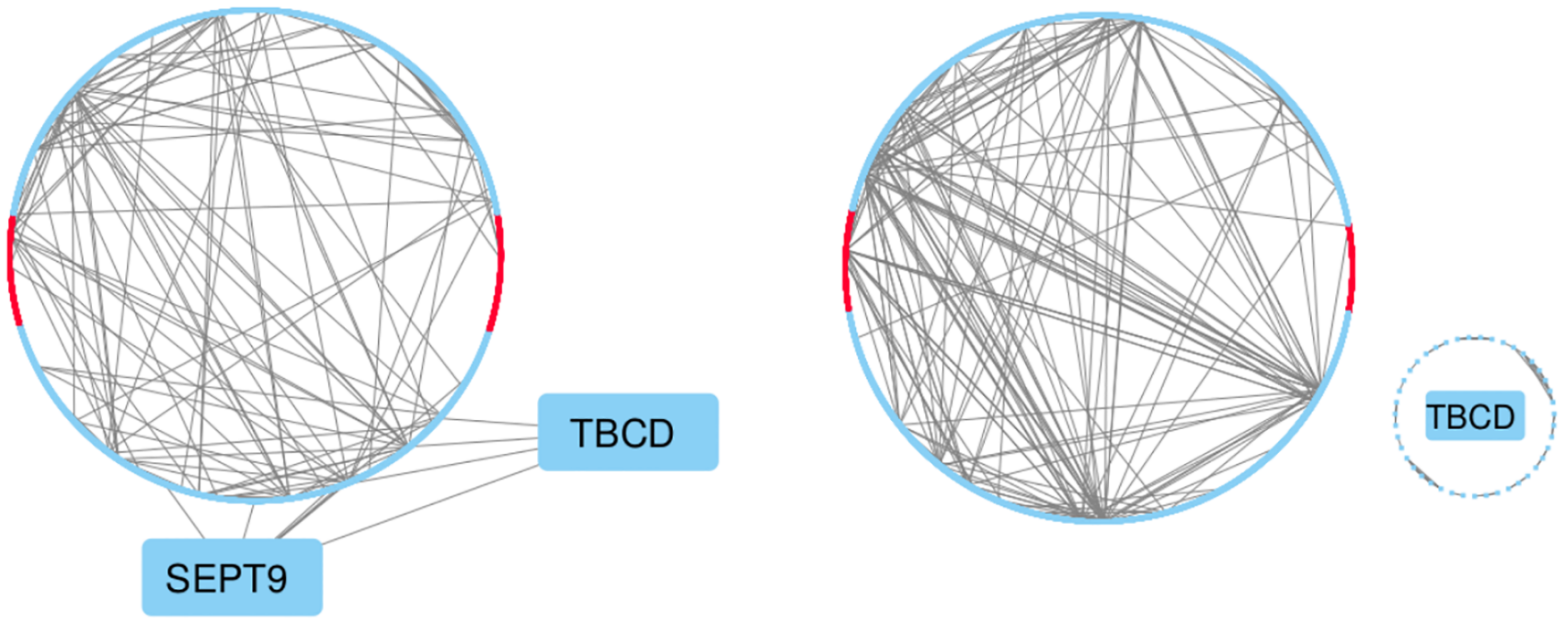
Estimated methylation networks for chromosome 17. The presence of uninformative short-range regional connections obtained with raw probe data (right) is illustrated via the TBCD gene. The region-based network (left) eliminates local connections allowing for longer range relationships (e.g. SEPT9 gene).

Results for the probe-based and region-based epigenetic networks are shown in Fig 6. Networks inferred for probe features contain a much larger proportion of edges connecting spatially adjacent probes; summaries of the genomic distances between connected nodes are given in Table 3. This finding is not unexpected given the high degree of spatial correlation inherent in methylation levels. Connections between local regions, however, are less meaningful biologically. SpaCC, on the other hand, reduces the data to its relevant biological units and hence inferred networks have a larger portion of long range connections that are more likely of interest to scientists. For example, the TBCD gene highlighted in Fig 6 plays a role in the cytokinesis stage of cell division [47] and has been shown to be upregulated in breast cancer [48]. As can be seen in the probe-based inferred network, probes in the TBCD region of chromosome 17 form only local connections. In contrast, SpaCC aggregates many of the probes in the regions surrounding the TBCD gene. The resulting network estimate no longer contains TBCD intra-gene connections, and instead forms longer range connections. One such long range connection illustrated above is to the SEPT9 gene region which has exhibited high expression levels in breast cancer cell lines [49]. While the connection between the epigenetics of TBCD and SEPT9 has not been experimentally established, both genes have been associated with breast cancer and thus represent the precise type of potential connection which scientists seek to discover using graphical models. By aggregating probes into relevant biological units, SpaCC-based graphical models are able to discover potentially novel functional relationships between epigenomic regions which would otherwise be masked.

**Table 3:** Summary of genomic distances between connected nodes for both SpaCC features and probe features, measured in kb. We note SpaCC favors longer range connections, as evidenced by larger genomic distance between neighbors.

Analysis Type	Mean	Median	25%	75%	90%	95%
Region	6554.18	30.33	.18	5252.73	31371.98	36738.76
Probe	2589.0428	0.075	.019	.208	2509.20	21967.56

### Region-based epigenetic-wide association studies

Finally, we study how SpaCC can improve systematic discovery of epigentic markers associated with disease through epigenome-wide association studies (EWAS). Analogous to more widely used GWAS, EWAS conducts univariate tests for association with an outcome at each epigenetic marker (probe at a CpG site) and adjusts significance levels for multiplicity. As the epigenome can encode environmental or behavioral characteristics of human subjects [50], EWAS can help discover how factors other than genetics contribute to disease. For EWAS with DNA Methylation data, however, the number of subjects is typically small relative to the ≈ 450,000 CpG sites measured by the latest Illumina platform, thus leading to a possibly under-powered study. To address this, we propose to first reduce methylation data to genomic regions via SpaCC and then conduct an association study on the regions; we term this a region-based epigenome-wide association study (rEWAS). As SpaCC retains genomic regions that behave as functional units, we expect that this will reduce the number of tests conducted, leading to an increase in statistical power, while still being able to detect epigenetic markers of disease. Note that testing regions in rEWAS is similar in spirit to testing groups of genetic markers based on linkage disequilibrium in GWAS [51]. Also note that some EWAS analyses have proposed to test regions, but they have typically considered tests for global methylation levels [52, 53] instead of localized genomic regions as we propose with SpaCC. In this section, we first evaluate the efficacy of SpaCC-based rEWAS via a simulation study and then present a rEWAS example to detect epigenetic markers of survival in lung cancer.

#### rEWAS simulation study

Since rEWAS is a new type of association study, we first use simulated methylation data to assess the performance of SpaCC-based rEWAS compared to traditional EWAS analysis using the raw probes. As in Section Simulation Studies: Methylation Region Detection, we base our simulated data on TCGA Breast Cancer Level 3 methylation data for chromosome 17. We obtain initial regions, {*c*_*g*_} via SpaCC and then simulate region means for each subject as *m*_*ig*_ ∼ *beta*(2, 2). Next, individual probes, *X*_*ij*_ are simulated as deviations about the region means via *X*_*ij*_ ∼ *beta*(*α*, *τ*), where *α*, *τ* are chosen to ensure *E*[*X*_*ij*_] = *m*_*ig*_. Finally, we generate our response as a linear function of a subset of the region means: $y_{i}=\beta_{1}m_{ig_{1}}+\ldots \beta_{d}m_{ig_{d}}+\epsilon_{i}$, where *ϵ* ∼ *N*(0, *σ*^2^), and $\beta_{l}∼N(\beta_{seed},\sqrt{\beta_{seed}})$. The difficulty of the simulation is controlled by the signal-to-ratio (SNR) level, here the size of *β*_*seed*_ relative to *σ*^2^. Our simulation consists of *n* = 94 patients relative to *p* = 23,515 probes.

We compare our SpaCC-based rEWAS approach to rEWAS using linkage disequilibrium [6] and the Fisher product method [54] as well as EWAS on the raw probes; see S3 Appendix for details. For all methods, we fit a univariate linear regression model at each probe or region and corrected for multiplicity using Benjamini-Hochberg’s method to control the FDR [55]. The objective is to recover the regions or all the probes contained within the regions that determine the outcome. Notice then that there are two ways to report the true positive rate (TPR) and false discovery proportion (FDP) for this simulation study. First, we can use the regions detected as significant and compare the spatial extent of a detected region to that of the corresponding true region to determine a true positive rate; we call this the region point-of-view (RegionPOV) and the FDP is defined analogously. Second, we can compare the probes detected, or the probes within regions detected as significant, to the probes that lie within the true regions to determine the true positive rate; we call this the probe point-of-view (ProbePOV). S4 Appendix contains formal definitions of these metrics used to evaluate our simulation study.

**Fig 7:**
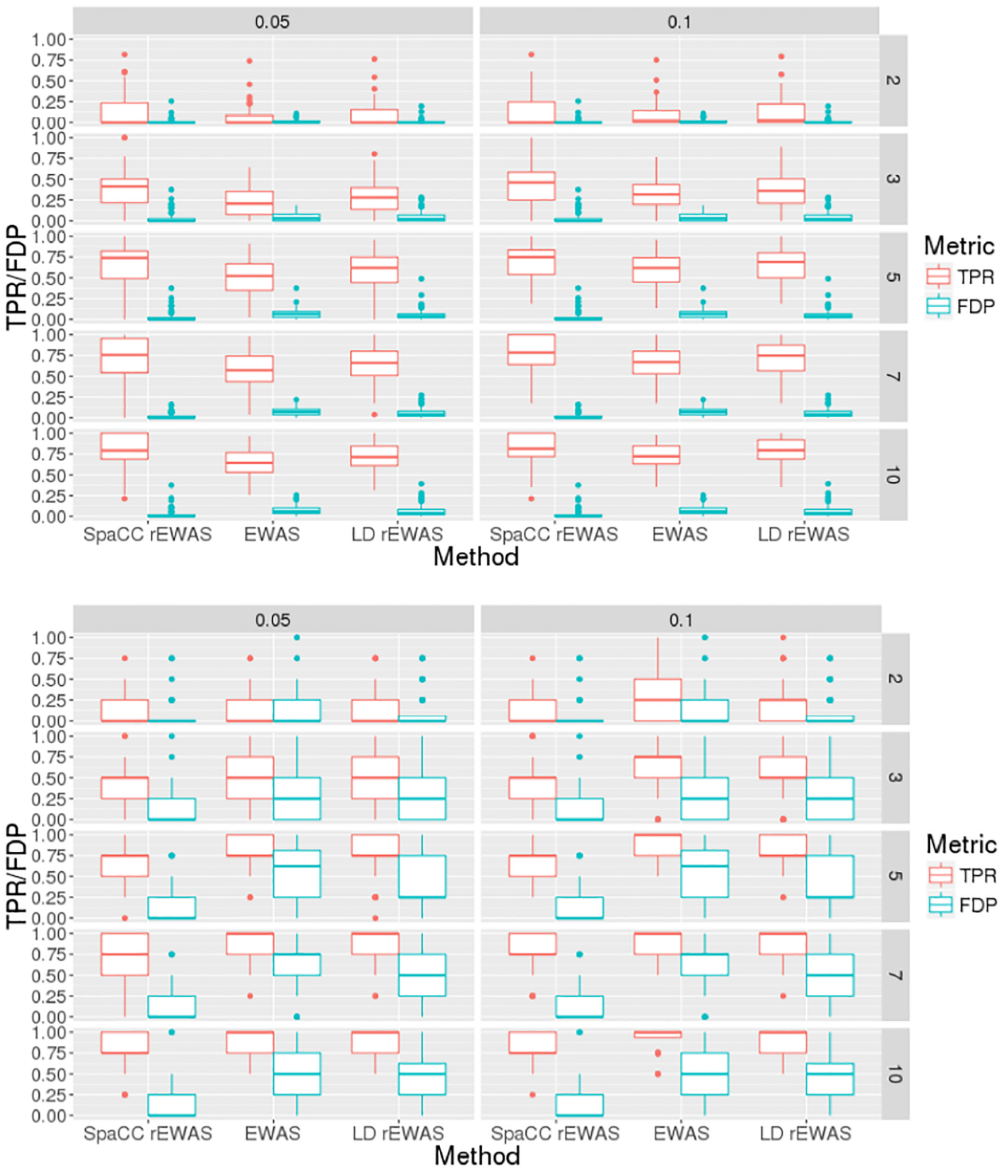
rEWAS simulation results: True positive rate (TPR) and false discovery proportion (FDP) for SpaCC-based rEWAS, LD-based rEWAS and probe-based EWAS over various SNR levels and FDR levels. Results are reported according to both probe point-of-view (ProbePOV; top) and Region point-of-view (RegionPOV; bottom) metrics.

In Fig 7, we report the results of our simulation study at various SNR levels and FDR levels. Compared to probe-based EWAS and LD-based rEWAS, SpaCC-based rEWAS, achieves comparable FDP levels while achieving higher TPR according to both probe and region-based metrics. Overall, this study confirms our intuition that first reducing methylation data to regions via SpaCC, leads to increases in statistical power for EWAS.

#### Lung cancer rEWAS study

**Fig 8:**
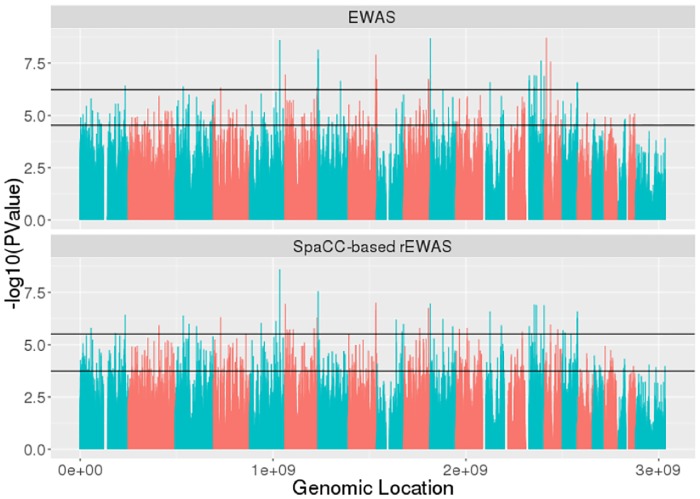
Manhattan plots with 1% and 5% FDR lines for EWAS (top) and SpaCC-based rEWAS (bottom) analysis for lung cancer methylation. We note the region-based analysis recovers a sizable portion of the probes deemed significant in the probe-based analysis. In addition, a large number of additional probes are recovered at an equivalent FDR-level.

We now use our SpaCC-based rEWAS approach to discover epigenetic markers associated with lung cancer survival. We use the TCGA lung cancer DNA methylation data which has *n* = 458 patients and *p* = 394,001 probes across all chromosomes [56]. A univariate Cox proportional hazards model is used to test for associations with survival at each probe (CpG site) for EWAS or at each SpaCC-region for rEWAS. The Benjamini-Hochberg procedure was used to control the FDR at the 1% and 5% levels. The p-values at each genomic location for EWAS and SpaCC-based rEWAS are displayed in Manhattan plots in Fig 8; the alternating colors represent chromosomes. Horizontal lines are shown at the 1% and 5% FDR levels; vertical lines denoting p-values that cross these thresholds are statistically significant. Notice that the Manhattan plots for both EWAS and rEWAS retain a similar shape, indicating that both methods found a common epigenome-wide signature for lung cancer survival. On closer inspection, we see that SpaCC-based rEWAS yields a larger number of discoveries at equivalent FDR-levels. At the 1% FDR level, EWAS found 29 significant CpG sites, whereas SpaCC-based rEWAS found 49 significant regions which contain a total of 77 CpG sites. Similarly at the 5% FDR level, EWAS had 287 discoveries while SpaCC-based rEWAS found 556 regions which contain 861 CpG sites. The overlap in significant discoveries are shown in Tables 4 and 5. Notice especially at the 1% FDR level that our SpaCC rEWAS method missed only 4 CpG sites declared significant by EWAS, but in the converse, EWAS missed 54 discoveries made by rEWAS.

**Table 4:** Probe (region) significance overlap between region and probe based analysis at 5% FDR level. Total Number of .05-level probes is 287. Total Number of .05-level regions is 556. Total Number of probes in .05-level regions is 861.

		Region	
		+	−
Probe	+	247 (216)	40 (39)
	−	614 (340)	

**Table 5:** Probe (region) significance overlap between region and probe based analysis at 1% FDR level. Total number of .01-level probes is 29. Total number of .01-level regions is 49. Total Number of probes in .01-regions is 77.

		Region	
		+	−
Probe	+	25 (21)	4 (4)
	−	52 (28)	

Our analysis reveals several important epigenetic markers which are largely consistent with the cancer literature. In the top portion of the Table 6, we list the most significant discoveries from rEWAS which were also discovered by EWAS. We also report the epigenetic marker location, its gene target or the nearest gene, and a brief description of its role in the cancer literature. Note that further details on the literature are given in S6 Appendix. In the bottom portion of Table 6, we highlight the most significant discoveries found exclusively by rEWAS; these are known markers for lung cancer. Overall, our analysis reveals that reducing methylation data to biologically meaningful genomic regions via SpaCC before conducting EWAS studies, leads to major increases in statistical power for discovering epigenetic markers.

**Table 6:** Significant discoveries found by SpaCC-based rEWAS. The top portion describes the top ten most significant discoveries; the bottom portion describes a subset of discoveries detected exclusively by rEWAS.

Gene	p-value	Chrm.(Loc.)	Description
LARP1	2.5e-9	5 (154.09–154.197)	Regulator of mTOR, prognostic marker
ZFAND2A	2.7e-8	7 (1.198–1.199)	Target for lung cancer therapy
TRAPPC9	9.8e-8	8 (140.74–141.46)	High expression in cancer cell lines
PKP3	1.0e-7	11 (.39–.40)	Oncogene, prognostic marker
GMDS	1.1e-7	6 (1.62–2.24)	Relation to NK escape
FBN1	1.1e-7	15 (48.70–48.93)	Hypermethylation in colorectal cancer
MYO1E	1.2e-7	15 (59.42–59.66)	Inhibition may prevent metastasis
IGF1R	1.2e-7	15 (99.19–99.50)	Silencing enhances sensitivity to DNA-damage
FAM53B	1.7e-7	10 (126.30–126.43)	Role in cell proliferation
ANAPC11	2.2e-7	17 (79.84–79.85)	Role in lung development.
CCDC12	7.6e-6	3 (46.96–47.02)	Contained in 3p21.3 tumor suppressor region
WWOX	1.6e-5	16 (78.13 -79.24)	Biomarker for lung cancer
ARL14	1.8e-5	5 (160.394–160.396)	Homologue to tumor suppressor gene ARLTS1

## Discussion

Building on the success of fusion-based penalties and convex clustering methods, we have introduced a clustering technique for spatially correlated data called Spatial Convex Clustering, or SpaCC. Our work has focused on SpaCC’s application to spatial genomics data, including copy number variation and methylation. While existing segmentation methods (both single- and multi-subject) exist for problem of Copy Number Segmentation, none currently employ the degree of automation found via SpaCC. With its ability to handle missing data and choose the number and extent of multi-subject genomic regions in a data-driven manner, SpaCC gives practitioners a powerful new tool for copy number analysis. More novel is SpaCC’s application outside the traditional realm of Copy Number Segmentation. Through both simulations and real data examples we have shown SpaCC to be a successful tool for reducing DNA methylation data to its functional genomic regions. This region-based approach to methylation data yields increased biomarker discovery along with an interpretable feature set. SpaCC’s success in the non-traditional domain of methylation data signals many potential applications across a variety of spatial settings. While this paper has focused on the analysis of methylation data, the SpaCC framework is not limited to this data type, nor genomic data in particular. Extensions of SpaCC may be appropriate for clustering spatially registered read counts from RNA-sequencing data to discover new isoforms and alternate splicing. Beyond genomics, similar fusion-based approaches may also be applied other biological data sources with known local spatial structure such as brain imaging. In all applications, SpaCC provides a automated data-driven approach to region detection, eliminating or reducing subjective or ad-hoc decisions. Taken together, the method and future extensions can allow scientists to perform a single integrative analysis to discover meaningful regions across differing biological modalities. An R package SpaCCr implementing our method is available from CRAN [57].

## Supporting information

S1 Appendix Spatial weight description. Description of weight choices for Copy Number and Methylation data.
(PDF)

S2 Appendix Clustering metrics. Description of various clustering metrics.
(PDF)

S3 Appendix Alternative region-based methods. Description of Linkage Disequilibrium and Fisher’s Method.
(PDF)

S4 Appendix Recovery points of view. Description of Probe Point of View (ProbePOV) and Region Point of View (RegionPOV).
(PDF)

S5 Appendix Copy number simulation. Description of data-based Copy Number simulation setup.
(PDF)

S6 Appendix Lung cancer rEWAS discoveries. Detailed citations for rEWAS discoveries.
(PDF)
