# Supplementary material for: Genomic region detection via Spatial Convex Clustering
Source: PLoS One. 2018 Sep 11;13(9):e0203007. doi: 10.1371/journal.pone.0203007 (PMC6133280; doi:10.1371/journal.pone.0203007)
Supplement: S1 Appendix — Description of weight choices for Copy Number and Methylation data. (PDF) [file pone.0203007.s001.pdf]

Appendix 1: Spatial Weight Description

Spatial weights are chosen in a biologically motivated fashion depending on data type. For both methylation and copy number data our weights are determined via $w_i = \exp\{-\sigma d_i\}$, where $d_i = \text{dist}(\text{probe}_i, \text{probe}_{i+1})$ is the distance in basepairs between probes. Here, σ is chosen based on properties of genomic data. For example with copy number data, it is common to have sizable portions of a chromosome amplified or deleted [1]. Hence, weights that decay at a slower rate as a function of genomic distance will perform well for CNV data; specifically, we take $\sigma = 0.00001$ in our empirical studies. For methylation data, we expect methylated regions to form in small localized CpG islands near promoter regions of genes [2]. Hence, we take $\sigma = 0.0002$ for methylation data, which yields a more rapid decay in weights as a function of genomic distance. We include a plot of weight value by genomic distance for each of our data types.

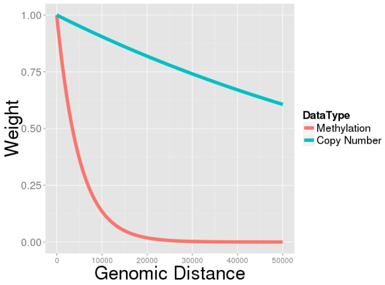

Fig 1. Example of spatial weights as a function of genomic distance for Methylation and Copy Number data. For Copy Number, weights decay more slowly as a function of genomic distance, allowing for the discovery of larger genomic segments. Methylation weights by constrast encourage shorter length segments.

References

1. Redon R, Ishikawa S, Fitch KR, Feuk L, Perry GH, Andrews TD, et al. Global variation in copy number in the human genome. nature. 2006;444(7118):444–454.

2. Eckhardt F, Lewin J, Cortese R, Rakyan VK, Attwood J, Burger M, et al. DNA methylation profiling of human chromosomes 6, 20 and 22. Nature genetics. 2006;38(12):1378–1385.
