# Supplementary material for: Genomic region detection via Spatial Convex Clustering
Source: PLoS One. 2018 Sep 11;13(9):e0203007. doi: 10.1371/journal.pone.0203007 (PMC6133280; doi:10.1371/journal.pone.0203007)
Supplement: S2 Appendix — Description of various clustering metrics. (PDF) [file pone.0203007.s002.pdf]

Appendix 2: Description of Clustering Metrics

Let $\Omega = \{1, 2, \ldots, p\}$ denote the set of probes to be clustered. For simulation studies we consider a partition of Ω, $\mathcal{C} = \{\mathcal{C}_l\}_{l=1}^{c}$, to act as ground truth. All methods considered will return a clustering/partition of Ω, denoted by $\hat{\mathcal{C}} = \left\{\hat{\mathcal{C}}_k\right\}_{k=1}^{d}$. The various clustering metrics will compare $\mathcal{C}$ and $\hat{\mathcal{C}}$, yielding a score which measures their agreement. We follow [1] in defining the various metrics below; see their paper for a fuller discussion.

Preliminaries Many clustering metrics partition the $\binom{p}{2}$ pairwise comparisions of elements in Ω into 4 sets. Let n_{11} denote the number of pairs which occur in the same cluster for both $\mathcal{C}$ and $\hat{\mathcal{C}}$. Let n_{00} denote the number of pairs which occur in different clusters for both $\mathcal{C}$ and $\hat{\mathcal{C}}$. Let n_{10} denote the number of pairs which occur in the same cluster for $\mathcal{C}$ but different clusters for $\hat{\mathcal{C}}$. Let n_{01} denote the number of pairs which occur in different clusters for $\mathcal{C}$ but the same cluster for $\hat{\mathcal{C}}$. Also useful is the cardinality of the intersection between two parition elements: $m_{lk} = \mid \mathcal{C}_l \cap \hat{\mathcal{C}}_k \mid$.

Rand Index The Rand Index between $\mathcal{C}$ and $\hat{\mathcal{C}}$ is given by

$$R(\mathcal{C}, \hat{\mathcal{C}}) = \frac{2(n_{11} + n_{00})}{p(p-1)}$$

Adjusted Rand Index The Adjusted Rand Index between $\mathcal{C}$ and $\hat{\mathcal{C}}$ is given by

$$R_{adj}(\mathcal{C}, \hat{\mathcal{C}}) = \frac{\sum_{l=1}^{c} \sum_{k=1}^{d} \binom{m_{lk}}{2} - t_3}{\frac{1}{2}(t_1 + t_2) - t_3}$$

with

$$\begin{aligned} t_1 &= \sum_{l=1}^{c} \binom{\mid \mathcal{C}_l \mid}{2} \\ t_2 &= \sum_{k=1}^{d} \binom{\mid \hat{\mathcal{C}}_k \mid}{2} \\ t_3 &= \frac{2 t_1 t_2}{p(p-1)} \end{aligned}$$

Jaccard Index The Jaccard Index is given by

$$J(\mathcal{C}, \hat{\mathcal{C}}) = \frac{n_{11}}{n_{11} + n_{10} + n_{01}}$$

Variation of Information Finally our entropy based metric, Variation of Information, is given by

$$VI(\mathcal{C}, \hat{\mathcal{C}}) = H(\mathcal{C}) + H(\hat{\mathcal{C}}) - 2I(\mathcal{C}, \hat{\mathcal{C}})$$

where

$$\begin{aligned} H(\mathcal{C}) &= -\sum_{l=1}^{c} P(l)\log_2 P(l) \\ I(\mathcal{C},\hat{\mathcal{C}}) &= \sum_{l=1}^{c}\sum_{k=1}^{d} P(l,k)\log_2\left(\frac{P(l,k)}{P(l)P(k)}\right) \\ P(l) &= \frac{\mid \mathcal{C}_l \mid}{p} \\ P(l,k) &= \frac{\mid \mathcal{C}_l \cap \hat{\mathcal{C}}_k \mid}{p} \end{aligned}$$

References

1. Wagner S, Wagner D. Comparing clusterings: an overview. Universität Karlsruhe, Fakultät für Informatik Karlsruhe; 2007.
