# Supplementary material for: Genomic region detection via Spatial Convex Clustering
Source: PLoS One. 2018 Sep 11;13(9):e0203007. doi: 10.1371/journal.pone.0203007 (PMC6133280; doi:10.1371/journal.pone.0203007)
Supplement: S3 Appendix — Description of Linkage Disequilibrium and Fisher’s Method. (PDF) [file pone.0203007.s003.pdf]

Appendix 3: Description of Alternative Region-based Methods

Linkage Disequilibrium The Linkage Disequilibrium (LD) method follows [1] by creating "methylation LD blocks". In particular CpG sites are coalesced into a block if their absolute correlation exceeds 0.3 and their genomic distance is less than 100bp; the block will continue to accrete CpG sites so long as these conditions are met. Once one of the conditions (either absolute correlation or genomic distance) fails to hold the block is ended and a new block is begun, proceeding in a similar manner. The resulting output is a partitioning of the CpG sites into blocks, or regions.

For both the methylation simulation studies of Section 3.2 and rEWAS simulation studies of Section 4.3.1 the output of both LD and SpaCC methods are evaluated identically, as they both return a partition of the CpG sites.

Linkage Disequilibrium + Fisher's Method Fisher's Method provides a method for p-value aggregation. In the case of rEWAS studies this provides an alternative method for calculating regional p-values. In particular, let $p_1, \ldots, p_j, \ldots, p_R$ denote p-values obtained via probe-wise regression. Further assume these probes/CpG sites belong to a common block/region as detected by the LD method described above. A test statistic for this region is calculated via

$$X^2 = -2 \sum_{j=1}^{R} ln(p_j)$$

and a p-value for this region is in turn calculated relative to a χ^2_{2R} distribution; as is the case for other region-based methods, the resulting regional p-values are subsequently corrected for multiple testing.

References

1. Shoemaker R, Deng J, Wang W, Zhang K. Allele-specific methylation is prevalent and is contributed by CpG-SNPs in the human genome. Genome research. 2010;20(7):883–889.
