# Supplementary material for: Genomic region detection via Spatial Convex Clustering
Source: PLoS One. 2018 Sep 11;13(9):e0203007. doi: 10.1371/journal.pone.0203007 (PMC6133280; doi:10.1371/journal.pone.0203007)
Supplement: S4 Appendix — Description of Probe Point of View (ProbePOV) and Region Point of View (RegionPOV). (PDF) [file pone.0203007.s004.pdf]

Appendix 4: Description of Recovery Points of View

In the rEWAS simulations of Section 4.3.1 we report both the True Positive Rate (TPR) and False Discovery Proportion (FDP) metrics for all methods. The calculation of both metrics requires knowledge of the ground truth, namely which regions or probes determine the response. Because both persepectives, either region or probe, are valid, we report two versions of each metric under the titles of Region Point of View (RegionPOV) and Probe Point of View (ProbePOV). Here we give details regarding the calculation of each metric.

We recall the typical TPR and FDP metrics are defined by

$$\begin{aligned} TPR &= \frac{\text{Number of True Discoveries}}{\text{Number of Positives}} \\ FDP &= \frac{\text{Number of False Discoveries}}{\text{Number of Discoveries}} \end{aligned}$$

Let $\Omega = \{1, \dots, p\}$ denote the set of probes. Let $\mathcal{C} = \{\mathcal{C}_l\}_{l=1}^{c}$ denote the true partition of Ω taken as ground truth. Let $\mathcal{C}_{Resp} \subset \mathcal{C}$ denote the regions associated with the response via $y = \beta_1 m_{l_1} + \cdots + \beta_R m_{l_R} + \epsilon$, with m_l denoting the mean measurement in region l. Finally let $\mathcal{P}_{Resp} = \{j \in \mathcal{C}_l \mid \text{ some } \mathcal{C}_l \in \mathcal{C}_{Resp}\}$ denote the probes contained within regions associated the response.

Probe-based Methods We note that probe-wise methods return a set of individual probes deemed significant discoveries: $\hat{\mathcal{P}}_{Resp} \subset \Omega$. For these methods the ProbePOV versions of TPR and FDP are given by

$$\begin{aligned} TPR_{ProbePOV} &= \frac{\mid \hat{\mathcal{P}}_{Resp} \cap \mathcal{P}_{Resp} \mid}{\mid \mathcal{P}_{Resp} \mid} \\ FDP_{ProbePOV} &= \frac{\mid \hat{\mathcal{P}}_{Resp} \cap \mathcal{P}_{Resp}^{C} \mid}{\mid \hat{\mathcal{P}}_{Resp} \mid} \end{aligned}$$

For RegionPOV metrics we define the following region estimate for probe based methods: $\hat{\mathcal{C}}_{Resp} = \Big\{\mathcal{C}_l \in \mathcal{C} \mid j \in \mathcal{C}_l \text{ for some } j \in \hat{\mathcal{P}}_{Resp}\Big\}$. Given this region estimate we define the RegionPOV versions of TPR and FDP as follows

$$\begin{aligned} TPR_{RegionPOV} &= \frac{\mid \hat{\mathcal{C}}_{Resp} \cap \mathcal{C}_{Resp} \mid}{\mid \mathcal{C}_{Resp} \mid} \\ FDP_{RegionPOV} &= \frac{\mid \hat{\mathcal{C}}_{Resp} \cap \mathcal{C}_{Resp}^{C} \mid}{\mid \hat{\mathcal{C}}_{Resp} \mid} \end{aligned}$$

Region-based Methods We note that region-based methods return a set of regions deemed significant discoveries: $\hat{\mathcal{C}}$. For these methods the ProbePOV version of TPR and FDP are given by

$$\begin{aligned} TPR_{ProbePOV} &= \frac{\mid \hat{\mathcal{P}}_{Resp} \cap \mathcal{P}_{Resp} \mid}{\mid \mathcal{P}_{Resp} \mid} \\ FDP_{ProbePOV} &= \frac{\mid \hat{\mathcal{P}}_{Resp} \cap \mathcal{P}_{Resp}^{C} \mid}{\mid \hat{\mathcal{P}}_{Resp} \mid} \end{aligned}$$

where $\hat{\mathcal{P}}_{Resp} = \Big\{ j \in \mathcal{C}_l \mid \text{ some } \mathcal{C}_l \in \hat{\mathcal{C}} \Big\}$. Similarly RegionPOV metrics are defined by

$$\begin{aligned} TPR_{RegionPOV} &= \frac{\mid \hat{\mathcal{C}}_{Resp} \cap \mathcal{C}_{Resp} \mid}{\mid \mathcal{C}_{Resp} \mid} \\ FDP_{RegionPOV} &= \frac{\mid \hat{\mathcal{C}}_{Resp} \cap \mathcal{C}^C_{Resp} \mid}{\mid \hat{\mathcal{C}}_{Resp} \mid} \end{aligned}$$

where $\hat{\mathcal{C}}_{Resp} = \Big\{ \mathcal{C}_l \in \mathcal{C} \mid j \in \mathcal{C}_l \text{ for some } j \in \hat{\mathcal{P}}_{Resp} \Big\}$.
