# Supplementary material for: Genomic region detection via Spatial Convex Clustering
Source: PLoS One. 2018 Sep 11;13(9):e0203007. doi: 10.1371/journal.pone.0203007 (PMC6133280; doi:10.1371/journal.pone.0203007)
Supplement: S5 Appendix — Description of data-based Copy Number simulation setup. (PDF) [file pone.0203007.s005.pdf]

Appendix 5: Description of Copy Number Simulation Setup

We base our simulation on real TCGA Level 2 Ovarian Copy Number data for chromosome 17 with $n = 456$ patients and $p = 7103$ probes. It is known that outliers may adversely affect the performance of segmentation routines. We utilize the popular `copynumber` package to detect and remove outlying probes via the winsorization procedure [1]. While the SpaCC procedure can properly handle the missing values induced by the removal of outlying elements, many other segmentation routines require some form of imputation. In order to ensure that the simulation setup is consistent across all methods, we perform traditional k-nearest neighbors imputation on the resulting data via the `impute` package [2].

Our simulation setup utilizes both a *series basis* subject and *segment basis* subjects. For the series basis subject, we choose a subject with exactly one copy number segment as detected by the `DNAcopy` package. For the segment basis subjects, we choose two subjects which deliever both ”Easy” and ”Hard” copy number segments again as detected by the `DNAcopy` package. Here ”Easy” refers to relatively few (seven) segment with longer length. ”Hard” segments are more numerous (nine) and are shorter in length. We plot the base series and both ”Easy” and ”Hard” segment series below.

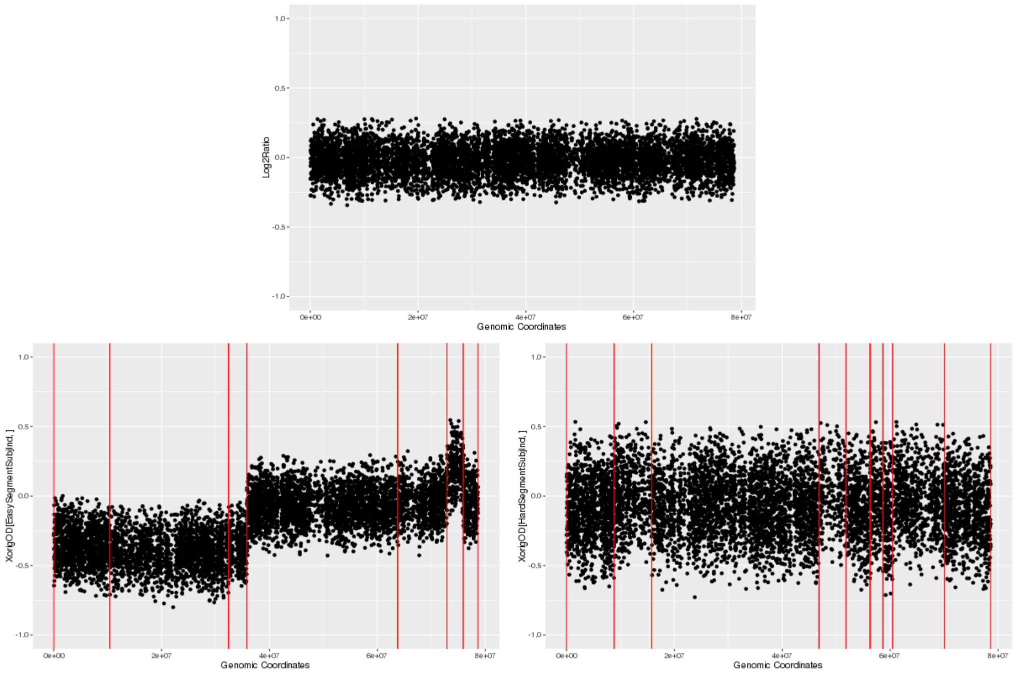

Fig 1. (Top) Series Basis subject with no copy number shifts detected. Simulation studies will shift this subject’s series. (Bottom Left) Easy Segment basis subject. (Bottom Right) Hard Segment basis subject.

The difficulty of simulations is controlled by the segment shape (Easy, Hard above) as well as the magnitude of the copy number shifts (Easy, Hard). We plot example

subjects under each of the simulation regiemes below.

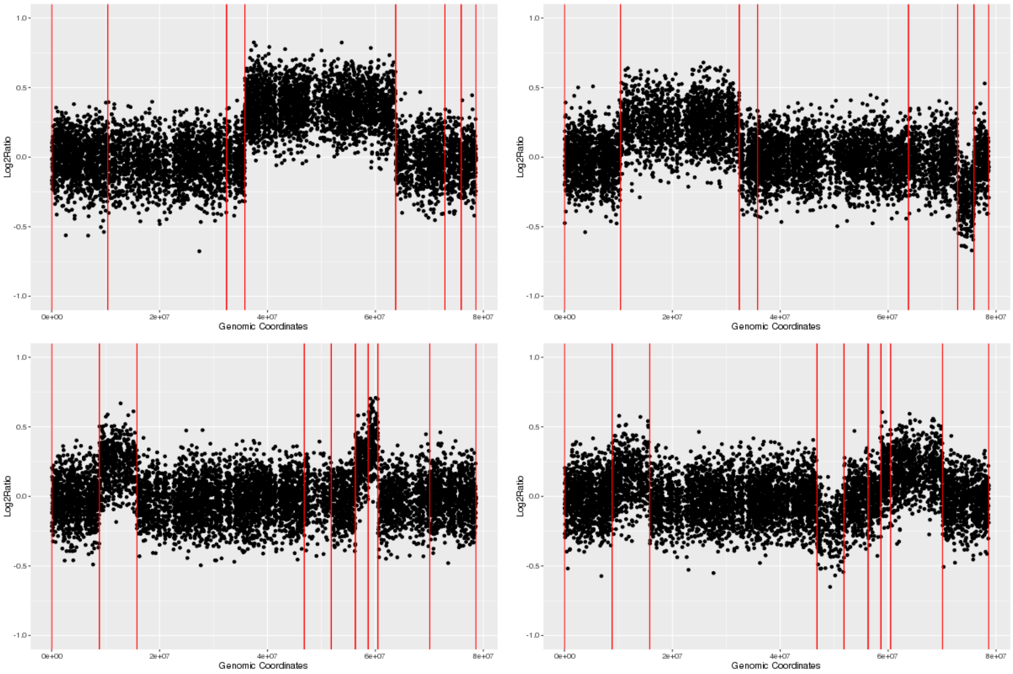

Fig 2. (Top Left) Easy Shape Easy Magnitidue. Few shifts with large changes. (Top Right) Easy Shape Hard Magnitude. Few shift with smaller changes. (Bottom Left) Hard Shape Easy Mangnitude. More shifts, with several occurring near to one another. (Bottom Right) Hard Shape Hard Magnitude. More shifts with small magnitude changes.

References

1. Nilsen G, Liestøl K, Van Loo P, Vollan HKM, Eide MB, Rueda OM, et al. Copynumber: Efficient algorithms for single-and multi-track copy number segmentation. BMC genomics. 2012;13(1):591.

2. Hastie T, Tibshirani R, Narasimhan B, Chu G. impute: impute: Imputation for microarray data;.
