# Supplementary material for: Genomic region detection via Spatial Convex Clustering
Source: PLoS One. 2018 Sep 11;13(9):e0203007. doi: 10.1371/journal.pone.0203007 (PMC6133280; doi:10.1371/journal.pone.0203007)
Supplement: S6 Appendix — Detailed citations for rEWAS discoveries. (PDF) [file pone.0203007.s006.pdf]

Appendix 7: Lung Cancer rEWAS Discoveries

Here we include a more detailed table of top rEWAS discoveries for Section 4.3.2.

Gene	p-value	Chrm.(Loc.)	Description	Literature
LARP1	2.5e-9	5 (154.09-154.197)	Regulator of mTOR, prognostic marker	[1]
ZFAND2A	2.7e-8	7 (1.198 - 1.199)	Target for lung cancer therapy	[2]
TRAPPC9	9.8e-8	8 (140.74 - 141.46)	High expression in cancer cell lines	[3]
PKP3	1.0e-7	11 (.39 - .40)	Oncogene,prognostic marker	[4]
GMDS	1.1e-7	6 (1.62 - 2.24)	Relation to NK escape	[5]
FBN1	1.1e-7	15 (48.70 - 48.93)	Hypermethylation in colorectal cancer	[6]
MYO1E	1.2e-7	15 (59.42 - 59.66)	Inhibition may prevent metastasis	[7]
IGF1R	1.2e-7	15 (99.19 - 99.50)	Silencing enhances sensitivity to DNA-damage	[8]
FAM53B	1.7e-7	10 (126.30 - 126.43)	Role in cell proliferation	[9]
ANAPC11	2.2e-7	17 (79.84 - 79.85)	Role in lung development.	[10]
CCDC12	7.6e-6	3 (46.96 - 47.02)	Contained in 3p21.3 tumor suppressor region	[11]
WWOX	1.6e-5	16 (78.13 -79.24)	Biomarker for lung cancer	[12]
ARL14	1.8e-5	5 (160.394 - 160.396)	Homologue to tumor suppressor gene ARLTS1	[13]

Table 1. Significant Discoveries found by SpaCC-based rEWAS. The top portion describes the top ten most significant discoveries; the bottom portion (in red) describes a subset of discoveries detected exclusively by rEWAS.

References

1. Xie C, Huang L, Xie S, Xie D, Zhang G, Wang P, et al. LARP1 predict the prognosis for early-stage and AFP-normal hepatocellular carcinoma. Journal of translational medicine. 2013;11(1):1.

2. Cao S. ZINC FINGER, AN1-TYPE DOMAIN 2A—A NOVEL TARGET FOR LUNG CANCER THERAPY. University of Pittsburgh; 2014.

3. Zhang Y, Liu S, Wang H, Yang W, Li F, Yang F, et al. Elevated NIBP/TRAPPC9 mediates tumorigenesis of cancer cells through NFκB signaling. Oncotarget. 2015;6(8):6160.

4. Furukawa C, Daigo Y, Ishikawa N, Kato T, Ito T, Tsuchiya E, et al. Plakophilin 3 oncogene as prognostic marker and therapeutic target for lung cancer. Cancer Research. 2005;65(16):7102–7110.

5. Moriwaki K, Noda K, Furukawa Y, Ohshima K, Uchiyama A, Nakagawa T, et al. Deficiency of GMDS leads to escape from NK cell-mediated tumor surveillance through modulation of TRAIL signaling. Gastroenterology. 2009;137(1):188–198.

6. Guo Q, Song Y, Zhang H, Wu X, Xia P, Dang C. Detection of hypermethylated fibrillin-1 in the stool samples of colorectal cancer patients. Medical Oncology. 2013;30(4):1–5.

7. Ouderkirk JL, Krendel M. Non-muscle myosins in tumor progression, cancer cell invasion, and metastasis. Cytoskeleton. 2014;71(8):447–463.

8. Rochester MA, Riedemann J, Hellawell GO, Brewster SF, Macaulay VM. Silencing of the IGF1R gene enhances sensitivity to DNA-damaging agents in

both PTEN wild-type and mutant human prostate cancer. Cancer gene therapy. 2005;12(1):90–100.

9. Thermes V, Candal E, Alunni A, Serin G, Bourrat F, Joly JS. Medaka simplet (FAM53B) belongs to a family of novel vertebrate genes controlling cell proliferation. Development. 2006;133(10):1881–1890.
10. Chan AH, Lee SM, Chim SS, Kok LD, Waye MM, Lee CY, et al. Molecular cloning and characterization of a RING-H2 finger protein, ANAPC11, the human homolog of yeast Apc11p. Journal of cellular biochemistry. 2001;83(2):249–258.
11. Agathanggelou A, Honorio S, Macartney DP, Martinez A, Dallol A, Rader J, et al. Methylation associated inactivation of RASSF1A from region 3p21. 3 in lung, breast and ovarian tumours. Oncogene. 2001;20(12):1509–1518.
12. Iliopoulos D, Guler G, Han SY, Johnston D, Druck T, McCorkell KA, et al. Fragile genes as biomarkers: epigenetic control of WWOX and FHIT in lung, breast and bladder cancer. Oncogene. 2005;24(9):1625–1633.
13. Yendamuri S, Trapasso F, Ferracin M, Cesari R, Sevignani C, Shimizu M, et al. Tumor suppressor functions of ARLTS1 in lung cancers. Cancer research. 2007;67(16):7738–7745.
